# Libel Suit in England

**Published:** 1892-06

**Authors:** 


### LIBEL SUIT IN ENGLAND.

The *Buffalo Medical and Surgical Journal* gives an account of the suit against Lawson Tait for making a statement to the husband of a former patient of Dr. Denholm as to the cause of death of the patient after an operation by Tait. The patient was suffering from a uterine myoma, and Dr. Delholm had employed Apostoli's electrical treatment without effect, further than accidentally to produce a vesical fistula, which added so much to the discomfort of the patient that she sought the advice of Mr. Tait in December, 1891. Mr. Tait condemned the electrical treatment, and advised an operation.

Two unsuccessful attempts had been made by Mr. Whitehead and Dr. L. Roberts to close the fistula. Mr. Tait said the fistula could be closed by an operation, but could not be done until after the myoma had been removed, owing to the great amount of inflammatory adhesions that existed, as a result of the electrical treatment.

The patient underwent the operation for the removal of the tumor, but succumbed from peritonitis within forty-eight hours. The husband was anxious to see Mr. Tait, but was unable to do so, but received the following letter, which contained the basis for the libel suit by Dr. Denholm:

My Dear Sir:--I saw your wife, with Mr. Christopher Martin, between two and three this morning, and matters were going on very fairly with her. At 7:30 I was summoned again, and found her sinking. You were summoned at once, and, I am glad to learn, reached her bedside before she died. The immediate cause of the unfortunate result is the rupture of a blood-vessel at or near the spot where the electrical needles caused so much damaging inflammation and sloughing. Had she never been submitted to that treatment, the case would have been a straightforward one, and her recovery almost certain.

Dr. Denholm construed Mr. Tait's letter to mean an unnessary charge against him; one wholly untrue, and demanded a *post mortem* examination, which was carried out by three eminent medical men, the result of which was to show that death was not due to hemorrhage, or a rupture of a blood-vessel, or to damaging inflammation, and sloughing consequent upon the previous electrical treatment, but to a peritonitis, the result of the operation.

The whole matter was finally settled by Mr. Tait having stated on the stand that his letter contained what he believed to be the truth, and he had nothing to retract, the law sustaining him as being "privileged."

Easy way to get out of a tight place; good thing to be "privileged." All hands then withdrew all "imputations," kissed, and made friends; that is, all but the poor woman, she was not present.

